# Effect of dominant follicle status at the time of retrieval on the clinical outcomes in natural cycle IVF combined with immature oocyte treatment

**DOI:** 10.18632/aging.204106

**Published:** 2022-06-07

**Authors:** Jian Hua Li, Tie Cheng Sun, Shui Wen Zhang, Ting Ting Jiao, Yan Bin Cheng, Pan Dong, Ri-Cheng Chian, Ye Xu

**Affiliations:** 1 Reproductive Medical Center, Senior Department of Obstetrics and Gynecology, The Seventh Medical Center of PLA General Hospital, Beijing 100700, China; 2 Reproductive Medical Center, Department of Obstetrics and Gynecology, Peking University International Hospital, Beijing 102206, China; 3 Center for Reproductive Medicine, Shanghai Tenth People’s Hospital of Tongji University, Shanghai 200072, China

**Keywords:** dominant follicle, *in vitro* maturation, natural cycle IVF, embryo culture, clinical outcomes

## Abstract

Objective: It is commonly believed that the oocytes from small follicles are unhealthy when a dominant follicle (DF) is recruited in the ovaries, especially when the DF is ovulated. This study aims to confirm whether the presence or ovulation of DF at the time of retrieval affects the clinical outcome of the natural cycle IVF with *in vitro* maturation (NC-IVF/M) treatment.

Methods: Data were collected from 446 women with regular menstrual cycle and 536 retrieval cycles using NC-IVF/M treatment. The cycles were divided into three groups based on the results of the oocyte retrieval cycle. Group A covers the collection of oocytes from the DF and small follicles; Group B incorporates failed oocyte retrieval from DF and then the oocytes are retrieved only from small follicles; and Group C includes the retrieval of oocytes only from small follicles accompanied with an ovulated DF. Furthermore, Group B and C have subgroups to include whether *in vivo* matured oocytes were obtained from small follicles. Following aspiration of DF and small follicles, mature oocytes were inseminated on the date of retrieval by intracytoplasmic sperm injection (ICSI) and the immature oocytes were matured *in vitro*. If the immature oocytes were matured *in vitro*, they were inseminated using ICSI, and then the embryos obtained from *in vivo* and *in vitro* matured oocytes were transferred accordingly.

Results: The oocytes from DF were successfully retrieved in 445 cycles (83.0%), failed to be retrieved in 54 cycles (10.1%) and ovulated in 37 cycles (6.9%). In Group A, an average of 2.0 ± 1.7 mature oocytes were retrieved, which was significantly higher than the average of Group B, with 1.3 ± 1.3 matured oocytes and Group C, with an average of 1.1 ± 1.5 matured oocytes (*P* < 0.01). However, the average number of immature oocytes retrieved from each group show no difference among the three groups. There was no significant difference in maturation rates of immature oocytes, fertilization rates among the three groups. The clinical pregnancy rate per transfer cycle is 34.5%, 34.6% and 25.7% in Group A, B and C, respectively. No significant differences were observed in embryonic development and implantation capacity in Group B and C in comparison to Group A. And there was no significant difference in clinical pregnancy, implantation, live birth and miscarriage rates among the three groups. No significant differences were observed in the developmental and implantation capacity according to with or without *in vivo* matured oocytes were retrieved in Group B and Group C.

Conclusion: The presence or ovulation of the dominant follicle from the ovaries does not significantly influence the developmental and implantation capacity of immature oocytes retrieved from small follicles, suggesting that NC-IVF/M is a promising treatment option for women without ovarian stimulation.

## INTRODUCTION

The world first *in vitro* fertilization (IVF) baby was born from natural cycle [1]. With the efficiency of IVF treatment, natural cycle was gradually replaced by ovarian stimulation cycle, as the success rate was directly related to the number of oocytes retrieved [2, 3]. Initially, the relatively simple and cheap medication, clomiphene citrate, combined with or without urinary gonadotrophins were used to stimulate ovaries [2]. Later, the use of gonadotrophin releasing hormone agonists (GnRH-a) was introduced in the mid-1980s to prevent premature luteinizing hormone surges [3]. The early 1990s introduced the development of highly purified and then recombinant gonadotrophins [4]. Gonadotrophin stimulation with GnRH analogues became the golden standard method to retrieve more oocytes [4, 5]. However, ovarian stimulation is always accomplished with direct or indirect side-effects, especially higher risk of ovarian hyper stimulation syndrome (OHSS), which is a life-threatening condition for women [4]. Therefore, many attempts with different efficiency on modified protocols were applied [6–8]. The dominant follicle (DF) selection and ovulation in mammals are complex and precisely regulated processes [9]. It is a common belief that only a dominant follicle is selected from a single cohort of antral follicles in the follicular phase of human menstrual cycle. The DF continues to develop and to ovulate while all other subordinate follicles regress [10–13]. However, it is demonstrated that the developmental competence of bovine oocytes from the small antra follicles is not adversely affected either by the presence of a dominant follicle or by the phase of folliculogenesis [14]. The serial transvaginal ultrasonographic evaluation revealed a wave phenomenon of follicular development where the small antral follicles in the luteal phase may not necessarily be in atresia but in the early stages of follicular development in women [15–17]. Interestingly, the natural cycle IVF combined with *in vitro* maturation (IVM) of immature oocytes (NC-IVF/M) treatment has been established as a vital treatment for different types of infertility [8, 18–20].

The dominant follicle can be distinguished from other follicles by its size of >10 mm in diameter in the natural cycle treatment [11]. It has been reported that the optimal timing of immature oocyte collection is based on the size of the DF in NC-IVF/M treatments [8, 21–24]. However, there are conflicting evidence regarding the importance of the DF on the day of aspiration before IVM. Some studies have shown a benefit in performing retrieval when the leading follicle reaches up to 10 mm in diameter [24, 25] whereas others indicated that there is a detrimental effect to the small follicles [22] and suggested that the treatment cycles should be cancelled [21, 26]. Therefore, the debate is ongoing on whether and how the sibling immature oocytes exposed to the selected DF could contribute to the overall pregnancy success [8, 21–23, 27–30] in unstimulated cycles, even though there is no difference observed in fertilization and embryo development [21, 22].

In clinical practice, the key point for natural cycle IVF/M treatment is the combination of oocyte from dominant follicle (DF) with the other oocytes from non-dominant follicles [8, 18, 19, 28–30]. This procedure, therefore, represents an interesting model for studying the development capacity from dominant and small follicles involved in the oocyte maturation process. In terms of the dominant follicles, whether or not ovulated when ultrasound scanning at retrieving, how to successfully retrieve the oocyte from DF, whether or not successfully retrieved were the focus we care for. For the oocyte from non-dominant follicles, the maturity and the rate of *in vitro* maturation (IVM) were the focus we concentrate on. Therefore, the pattern of oocyte from DF and SF at the time of retrieval is closely related to the development capacity of resulted embryos and the clinical outcomes after embryo transfer (ET). However, little is known about how the pattern of DF in clinical practice actually influences the development capacity of oocytes from small follicles even though after ovulation and the subsequent clinical outcomes in detail. Therefore, the real place for it has yet to be defined as we lack information for the oocytes from the small follicles when the dominant follicle was selected and ovulated in natural cycle IVF/M treatment.

In this study, we were to confirm the efficiency of natural cycle IVF/M treatment and to present the primary evidence of oocytes from the small follicle on the subsequent embryonic development capacity and clinical outcomes under the different patterns of dominant follicle in natural cycle IVF/M treatment in a large group.

## MATERIALS AND METHODS

### Patients

A total of 536 oocyte pickup (OPU) cycles were performed for 465 patients in this study. NC-IVF/M treatment cycles were performed on patients with at least 2 years of infertility, from April 2005 to December 2019. All women had normal ovaries, uterus and regular menstrual cycles. Patients diagnosed with polycystic ovary syndrome (PCOS) [4] were not included from this study. The mean age of the patients was 31.5 ± 4.1 years old. The study was approved by the Hospital Institutional Review Board and the written informed consent was obtained from all patients.

### Natural cycle IVF/M treatment

The treatment procedure for natural cycle IVF/M was performed as described previously [8, 18, 19]. In brief, a baseline transvaginal ultrasound scan on day 2 or 3 of the menstrual cycle was initiated and more than seven small antral follicles in both ovaries were evaluated. The ultrasound scans were repeated on day 7–9. When the dominant follicle reached 12–14 mm in diameter and the endometrial thickness was more than 6 mm, the patients were injected 10,000 IU human chorionic gonadotropin (hCG, Choragon, Ferring Pharmaceuticals, Mexico) intramuscularly.

After 36–38 hours after hCG injection, oocyte retrieval was performed using transvaginal ultrasound-guided aspiration. For the DF aspiration, 17-gauge double lumen needle (COOK, Eight Mile Plains, Queensland, Australia) was connected to a portable pump with a vacuum pressure of <100 mmHg. For small follicles, a 19-gauge single lumen needle (COOK) with a pressure <40 mmHg was used. The aspirated follicular fluid was collected in 10 mL tubes containing Quinn’s Advantage Medium w/HEPES with 2U/mL of sodium heparin. The follicular aspirates for small follicles were filtered with a Cell Strainer (Ø70μm, Falcon, Becton Dickinson and Company, NJ, USA), and washed three times with heparinized Quinn's Advantage Medium with HEPES to collect the cumulus-oocyte complexes (COCs). The oocyte maturity of COSs from the DF and small follicles were assessed under a stereo-microscope.

### *In vitro* fertilization (IVF) and *in vitro* maturation (IVM)

**Figure 1:**
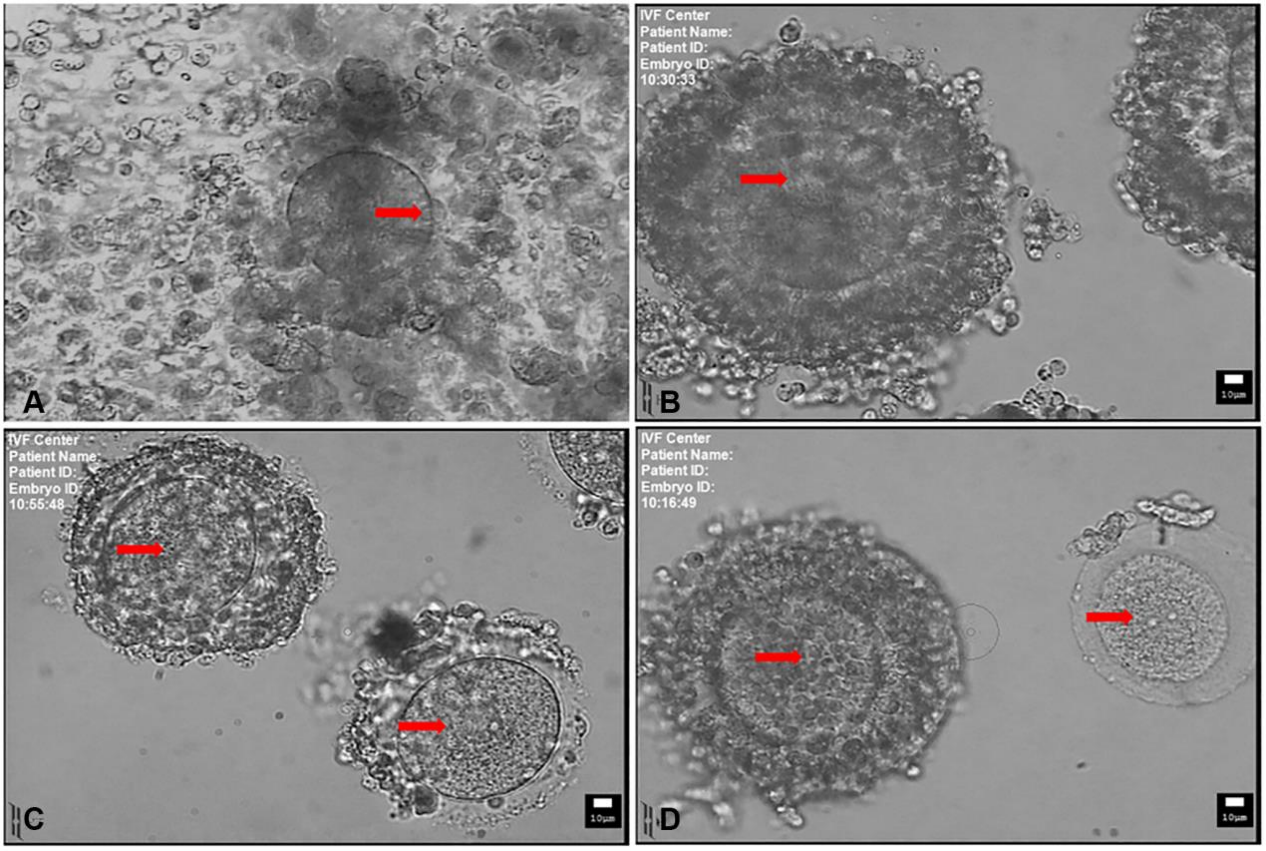
**Mature and immature oocytes collected at the time of retrieval.** (**A**) MII-stage oocyte with a disperse cumulus cells surrounding. Arrow indicates the first polar body. (**B**) Immature oocyte with compacting cumulus cells. (**C**) Immature oocyte with sparse cumulus cells. (**D**) Immature oocyte with compact (left) and denuded cumulus cells (right). Arrow indicates the germinal vesicle in (**B**, **C** and **D**). Scale bar: 10 μm.

The mature oocytes were inseminated 2 or 3 hours later by intracytoplasmic sperm injection (ICSI). The immature oocytes at Metaphase-I (MI) and germinal vesicle (GV) stages (Figure 1) were cultured in 1 mL maturation medium to induce final oocyte maturation at 37°C in 6% CO_2_, 5% O_2_ and 89% N_2_ with high humidity. The IVM culture medium contained 30% serum of the patient’s own (inactivated at 56° for 30 min) with 75 mIU/mL follicle stimulating hormone (FSH, Serono, Switzerland), 10 mIU/ml human menopausal gonadotrophin (Livzon Medical Groups; Zhu Hai, China) and 10 ng/ml recombinant human epidermal growth factor (Invitrogen, Carlsbad, CA, USA). The maturity of MI stage oocytes was re-evaluated after 3 hours IVM culture and the matured oocytes were subjected to ICSI 2 hours later. After 24 to 48 hours of IVM, if the oocytes mature, they were then inseminated by ICSI.

Following 16–18 h after ICSI, fertilization was evaluated by the appearance of 2 distinct pronuclei and 2 polar bodies. The zygotes were cultured in 20 μL droplets of G1-PLUS medium (Vitrolife, Gothenburg, Sweden) covered with paraffin oil (Vitrolife) and incubated according to standard procedures for further development.

### Embryo transfer and endometrial preparation

Embryo transfer (ET) was performed on day 3 after ICSI using an Ultrasoft Frydman catheter set (Laboratorie CCD, France) with echogenic guide. The endometrial preparation was started by the administration of 6 mg Estradiol Valerate (Delpharm Lille SAS, France) once daily from the day of oocyte retrieval. Luteal support was initiated with 100 mg progesterone in oil (Solvay Pharmaceuticals B.V., Weesp, Netherlands) daily on the day of ICSI procedure. On day 15 or 16 following oocyte retrieval, the level of serum ββ-hCG was tested to determine the pregnancy and clinical pregnancy was confirmed by the appearance of a gestational sac and fetal heart beat on ultrasound scan 6 weeks after ET.

### Group design

Natural cycle IVF/M procedures provide us a good model and allow us to distinguish oocytes from dominant follicle and small follicles in a same cycle and to characterize dominant follicle selection on the developmental capacity of embryos from small follicles under different situations.

**Figure 2:**
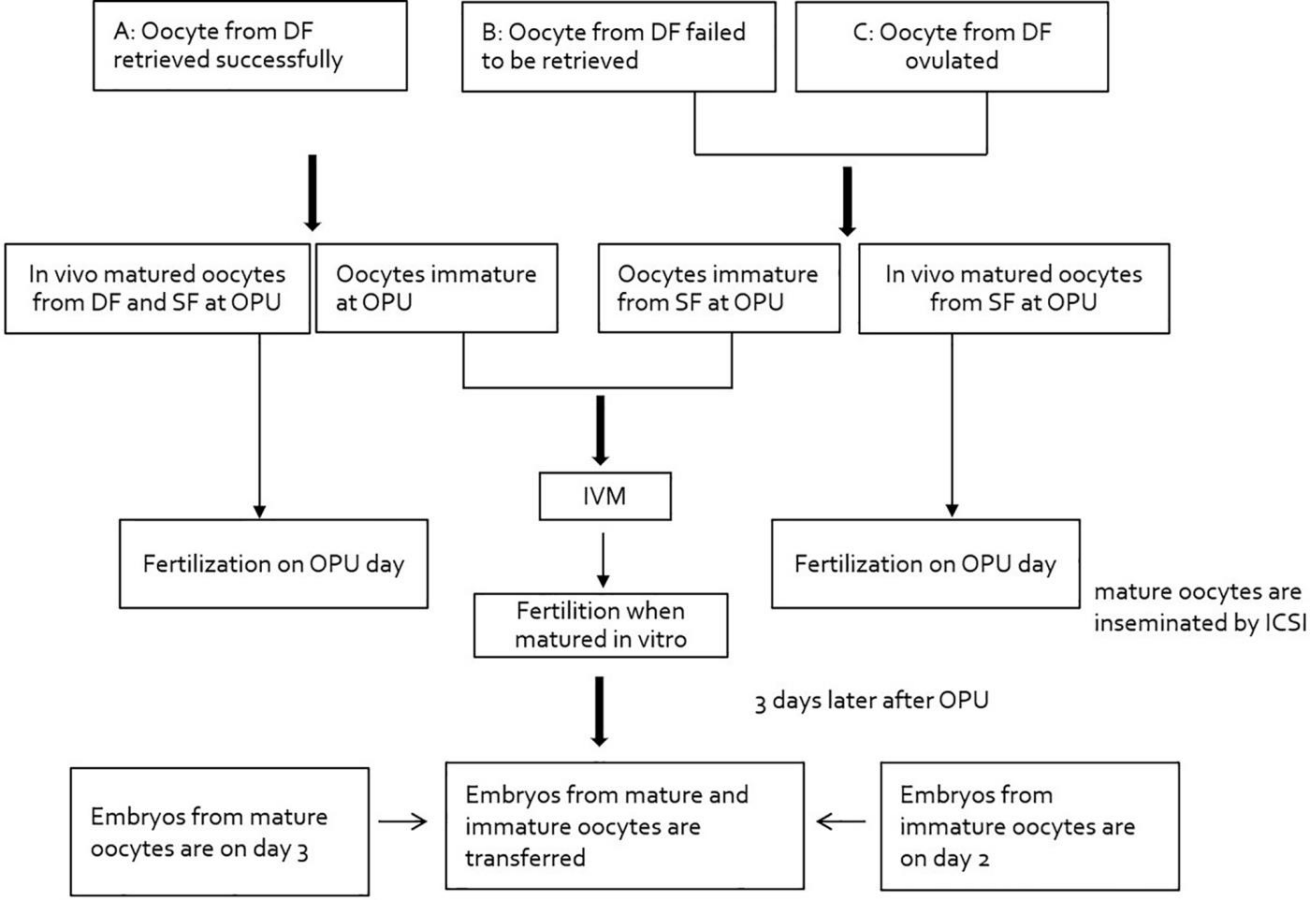
**Study flowchart: pattern of dominant follicle at the time of retrieval.** Abbreviations: DF: dominant follicle; OPU: oocyte pickup; ICSI: intracytoplasmic sperm injection.

Firstly, based on whether the oocyte from dominant follicle was retrieved or not from the DF at the time of retrieval, the treatment cycles were divided into 3 groups: Group A covers the successful retrieval of oocyte from the DF at the time of retrieval, with oocytes from small follicles; Group B includes the failed retrieval of oocytes from the DF at the time of oocyte pickup and the oocytes were then retrieved only from small follicles; Group C consists of DF that had ovulated at the time of retrieval and oocytes were retrieved only from small follicles (Figure 2). Furthermore, Group B and C were classified with B1 and B2, C1 and C2 with respect to whether *in vivo* mature oocytes were obtained small follicles or not, respectively.

### Statistical analysis

Statistical analysis was performed using the SPSS 20.0 software (SPSS Inc., Chicago, IL, USA). Comparison of frequency data between groups, such as clinical pregnancy, implantation, live birth, and miscarriage rates were performed by chi-square test. The non-paired *t*-test and Mann–Whitney test were applied to compare mean numbers. For other quantitative comparisons, analysis of variance (ANOVA) was used. A *p*-value below 0.05 was considered to be statistically significant.

## RESULTS

In total, 536 cycles (465 patients) were enrolled to perform NC-IVF/M treatment in this study. The basal characteristics and hormone levels of the three groups were shown in Table 1, no significant differences were found between groups. As shown in Table 1, the oocytes were successfully retrieved from the DF in 377 women with 445 cycles (Group A), in which was 83.0% of the treatment cycles. In Group B, there were 54 cycles (10.1% of the total treatment cycles) which failed to obtain the oocytes from the DF at the time of retrieval. In Group C, there were 37 cycles (6.9% of the total treatment cycles), in which oocytes from DF had ovulated from 34 patients at the time of retrieval. The mature oocytes can be retrieved from the DF but also from the small follicles (Figure 2).

**Table 1:** Comparison of clinical outcomes based on the retrieval or ovulation of DF at the time of retrieval with natural cycle IVF/M treatment.

**Variable**	**A**	**B**	**C**	***P***
No. of patients	377	54	34	
Age of women, years	31.2 ± 4.0	30.9 ± 3.8	31.5 ± 4.1	0.803
FSH (mIU/mL)	5.72 ± 2.45	6.27 ± 2.55	6.23 ± 2.43	0.094
LH (mIU/mL)	3.20 ± 2.14	2.95 ± 2.33	3.09 ± 2.53	0.347
Estradiol (pg/mL)	41.59 ± 17.29	42.31 ± 21.69	41.09 ± 16.81	0.982
Progesterone (ng/mL)	0.55 ± 0.35	0.49 ± 0.2	0.56 ± 0.33	0.598
No. of oocyte retrieval cycles (%)	445 (83.0)	54 (10.1)	37 (6.9)	
No. of completed ET cycles	441	52	35	
No. of oocytes retrieved	4757 (10.7 ± 5.1)	501 (9.3 ± 4.7)^*^	354 (9.6 ± 5.1)	0.036
No. of oocytes retrieved from DF	465 (1.0 ± 0.2)	–	–	
No. of mature oocytes retrieved	**879 (2.0 ± 1.7)**	69 (1.3 ± 1.3)^*^	**39 (1.1 ± 1.5)** ^*^	**<0.001**
No. of immature oocytes retrieved	3673 (8.3 ± 4.8)	408 (7.6 ± 4.3)	298 (8.1 ± 4.3)	0.528
Maturation rate	63.8 (2345/3673)	65.4 (267/408)	64.8 (193/298)	0.788
Fertilization rate	87.5 (2670/3052)	84.2 (266/316)	85.3 (191/224)	0.178
No. of embryos transferred	**1246 (2.8 ± 0.7)**	137 (2.5 ± 1.0)^*^	**98 (2.7 ± 1.3)**	**0.038**
Clinical pregnancy rate/ET cycle	34.5 (152/441)	34.6 (18/52)	25.7 (9/35)	0.571
Embryo implantation rate	15.2 (190/1246)	16.1 (22/137)	10.2 (10/98)	0.377
Cumulative live birth rate	69.1 (105/152)	66.7 (12/18)	66.7(6/9)	0.969
Singleton	88 (83.8)	10 (83.3)	6 (100.0%)	0.562
Twin	16 (15.2)	2 (16.7)	0	
Triplets	1 (1.0)	0	0	
Miscarriage rate/clinical pregnancy	27.0 (47/152)	33.3 (6/18)	33.3 (3/9)	0.969

Values are mean ± SD unless otherwise specified. Abbreviation: DF: dominant follicle. ^*^Significant difference compared with Group A. Abbreviations: No: number; FSH: follicle-stimulating hormone; LH: luteinizing hormone.

The mean number of oocytes retrieved from Group A was significantly higher than that of Group B (*P* < 0.05) with no difference to the mean of Group C (*P* > 0.05). In Group A, an average of 2.0 ± 1.7 *in vivo* matured oocytes were retrieved, which was significantly higher than the averages in Group B (1.3 ± 1.3) and Group C (1.1 ± 1.5) (*P* < 0.01). However, the average of immature oocytes retrieved showed no differences as well as the rates of *in vitro* maturation and fertilization among three groups. The clinical pregnancy rates per transfer cycle were 34.5%, 34.6% and 25.7% respectively for Group A, B and C. There was no significant difference in clinical pregnancy, implantation, live birth and miscarriage rates among the three groups.

Table 2 demonstrates the clinical outcomes based on whether the mature oocytes were retrieved from the small follicles in Group B and Group C. No differences were observed in the rates of *in vitro* maturation, fertilization and the cleavage rate no matter with or without *in vivo* matured oocyte from small follicles in Group B and C. Also, there were no significant differences were observed for clinical pregnancy rates, implantation rates, live birth rates as well as miscarriage rates among between the subgroups of Group B and Group C. However, live birth rates of 40.0% in Group B and 60.0% in Group C were reduced with the retrieval of only immature oocytes from small follicles as opposed to the rates of 76.9% and 75.0% in Group B and Group C, respectively, with the retrieval of *in vivo* matured oocytes from small follicles. The miscarriage rates of 60.0% for Group B and 40.0% for Group C without the retrieval of mature oocytes were higher than the miscarriage rates of 23.1% and 25.0% with 23.1% and 25.0% with the retrieval of mature oocytes for Group B1 and Group C1, respectively.

**Table 2:** Comparison of clinical outcomes based on whether or not mature oocytes were retrieved at the time of egg retrieval in Group B and C.

**Variable**	**B: oocyte failed from DF at retrieval**			**C: DF ovulated at retrieval**		
	**B1**	**B2**	***P***	**C1**	**C2**	***P***
No. of oocyte retrieval cycles	33	21		20	17	
No. of ET cycles	33	19		20	15	
Age of women, years	30.8 ± 38	31.1 ± 4.0	0.833	31.5 ± 3.1	31.5 ± 5.1	0.988
No. of oocytes retrieved	10.6 ± 5.1	7.2 ± 3.1	0.008	11.0 ± 5.7	7.9 ± 3.8	0.073
No. of mature oocytes retrieved	2.1 ± 1.0	–	–	2.0 ± 1.6	–	
No. of immature oocytes retrieved	8.2 ± 4.9	6.6 ± 3.1	0.210	8.56 ± 4.7	7.5 ± 3.8	0.453
Maturation rate *in vitro*	63.2 (170/269)	69.8 (97/139)	0.185	61.4 (105/171)	69.3 (88/127)	0.159
Fertilization rate	86.6 (187/216)	79.0 (79/100)	0.086	83.5 (111/133)	87.9 (80/91)	0.356
No. of embryos transferred	2.7 ± 1.0	2.6 ± 0.7	0.741	3.0 ± 1.1	2.5 ± 1.1	0.222
Clinical pregnancy rate/ET cycle	39.4 (13/33)	26.3 (5/19)	0.340	20.0 (4/20)	33.3 (5/15)	0.615
Embryo implantation rate	17.0 (15/88)	14.3 (7/49)	0.673	8.3 (5/60)	13.2 (5/38)	0.670
live birth rate/clinical pregnancy	76.9 (10/13)	40.0 (2/5)	0.352	75.0 (3/4)	60.0 (3/5)	0.633
Miscarriage rate/clinical pregnancy	23.1 (3/13)	60.0 (3/5)	0.352	25.0 (1/4)	40.0 (2/5)	0.633

Values are mean ± SD unless otherwise specified. Abbreviation: DF: dominant follicle. B1, C1: cycles with mature oocytes were retrieved from small follicles and embryos derived from oocytes matured *in vivo* and *in vitro*; B2, C2: cycles with only immature oocytes were retrieved from small follicles and embryos derived from oocytes matured *in vitro*.

## DISCUSSION

The present study displays a clear model of dominant follicle at the retrieval time in natural cycle IVF/M treatment and demonstrates that the clinical outcomes of NC-IVF/M were not significantly influenced by the retrieval of oocyte from dominant follicle or the DF ovulation at the time of retrieval. Follicle selection has been documented to occur only once from a single cohort of antral follicles in the early- to mid- follicular phase of the menstrual cycle, and then to develop and to ovulate [11]. The selection of a dominant follicle is generally believed to suppress the development of subordinate follicles and to initiate the atresia of the small antral follicles. However, more recent research suggests that selection may occur more than once in approximately one-quarter of apparently healthy women [15]. To date, most of the understanding of folliculogenesis and ovulation have come from animal models, especially from domestic animal and primate species [14, 31–33]. Few studies have investigated the exact role of dominant follicle on the oocytes from cohort of small follicles during human follicular and luteal phases in detail. The introduction of IVM into natural cycle IVF not only widens the possibilities of oocyte source but also provides us opportunity with clinical outcomes and a good model to characterize the significance of dominant follicle selection on the small antral follicles during the ovulatory process. In contrast to the notion of a single dominant follicle selection from the recruited wave, it has been documented that two or three waves of antral follicle recruitment were recognized during each estrous cycle, where a group of antral follicles begin to grow simultaneously at regular intervals [15, 16]. The new wave model for folliculogenesis in ovaries provided better opportunities for women to initiate flexible approach to IVF programs in the late follicular or luteal phase. The present results of oocytes from the cohort of small follicles after the dominant follicle is ovulated may support this notion that these oocytes came from newly developed follicles that emerged in a wave of luteal recruitment [34–37]. Although the total number of mature oocytes retrieved in Group A was significantly higher than that of Group B and Group C, comparable yields were observed in the rates of oocyte maturation, fertilization and cleavage among these three groups. These results are similar to the other literatures in IVM cycle [21–23]. Most of the literatures were focused on the effects of the size of the DF and maturity of oocytes at retrieval on the development potential and/or clinical outcomes in natural cycle IVF /M treatment [14, 30–32]. Moreover, only few studies compared the laboratory parameter between follicular phase and luteal phase [23–26]. Laboratory parameters of IVM procedure from the follicular and the luteal phase were conducted for fertility preservation of women with cancer in urgent situations except that of Pongsuthirak and Vutyavanich [35]. Therefore, there is a dramatic lack of data on the clinical outcome of the cryopreserved oocytes or the resultant embryos for these patients. And the clinical outcomes from nondominant follicles after ovulation of the dominant follicle were reported only in few cases for women in IVF/M cycle [8, 19, 38].

Nondominant small follicles have been reported to be a promising supplementary source of *in vivo* matured oocytes and the use of oocytes from those nondominant small follicles may increase the live birth rate in natural cycle IVF [38]. However, only women who had the retrieval of the DF combined with small follicles were included in their analysis and patients whose oocyte from the DF was not retrieved and ruptured were excluded in the study [38]. Therefore, the exact role of dominant follicle on the cohort of small follicles during the follicular development and ovulation in a regular menstrual cycle was not illustrated in detail and remains unclear. The present study is the first to report clinical outcomes of the oocytes derived from small follicles, especially after the ovulation of dominant follicle. As shown in the results of Table 1, because of the oocytes collected from dominant follicles, the mean number of *in vivo* matured oocytes and the total numbers of oocytes in Group A was significantly higher than that in Group B and C. However, no significant differences were observed for clinical pregnancy rates, implantation rates, live birth rates as well as miscarriage rates among these three groups. Even though the number of embryos transferred in group A with oocyte from DF retrieved was higher than that in group B with the failure of oocyte from DF retrieval, the clinical pregnancy rate was still not as high as that in group B. This just shows that DF or the number of mature oocytes at the time of oocyte retrieval did not affect the clinical pregnancy rate and further illustrates the developmental potential of oocytes from small follicles and that *in vitro* maturation of immature oocytes can yield comparable clinical pregnancy rates. Meanwhile, the present clinical pregnancy (25.7%) and implantation (10.2%) rates were much higher when DF had ovulated than the rates when the DF diameter was > 14 mm (17.1% and 4.9%, respectively) in the study of Son et al. (2008, 22). Despite the failure of oocyte retrieval from DF or the ovulation of DF, our results indicate that the healthy oocytes with the maturational and developmental competence could be yielded from the nondominant follicles and could become competent for implantation and live birth. Surprisingly, mature oocytes can also be retrieved from subordinate small follicles when the DF is not retrieved or ovulated in both Group B and C (Table 2). Accordingly, the pregnancy rate of 39.4% with *in vivo* matured oocytes was higher than the rate of 26.3% of only immature oocytes retrieved in Group B. The mature oocytes from nondominant follicles increased the number of both good-quality blastocysts and resulted in live births with no oocyte from the DF in modified natural cycle IVF [38]. The nondominant follicle-derived matured oocytes yielded 22.1% blastocysts and 10.5% clinical pregnancy rate. And the DF derived-matured oocytes yielded 52.6% blastocysts and 24.7% clinical pregnancy rate in their study. From our results, when the oocyte was failed to be obtained from the DF or ovulated from DF, the immature oocytes were also a promising source in natural cycle IVF treatment, which produced higher clinical pregnancy than only matured oocytes retrieved from nondominant and dominant follicles.

The present results of clinical pregnancy rate (26.3%) from the retrieval of only immature oocytes in Group B was comparable to 9.1% [30] and 34.5% [28] of the retrieval of mature oocytes from both DF and small follicles. The embryos from *in vitro*–matured oocytes were diagnosed as chromosomally normal for the chromosome analysis as from *in vivo*–matured oocyte in natural cycle IVF/M treatment [39]. These results demonstrate that immature oocytes from the cohort of small follicles are a promising source as they can not only produce healthy mature oocytes following IVM [40] but can contribute to the overall pregnancy success when exposed to dominant follicle of any conditions. Therefore, it is recommended to continue to retrieve oocytes from small follicles even with an ovulated DF on retrieving day in NC-IVF/M treatment.

In conclusion, developmentally competent oocytes as well as comparable clinical yields could be produced from subordinate follicles in any phase of the menstrual cycle when a significant dominant follicle has developed or ovulated. These demonstrate that natural cycle IVF/M is a promising alternative for infertility treatment. Furthermore, with the opportunity for IVM to be improved and optimized, the complete development of an immature oocyte is to be ensured in the future. In addition, the pregnancy outcome and neonatal data in natural cycle IVF/M still needs to be classified.
